# Helper-free Production of Laboratory Grade AAV and Purification by Iodixanol Density Gradient Centrifugation

**DOI:** 10.1016/j.omtm.2018.05.001

**Published:** 2018-05-08

**Authors:** Sean M. Crosson, Peter Dib, J. Kennon Smith, Sergei Zolotukhin

**Affiliations:** 1 Department of Pediatrics, Division of Cell and Molecular Therapy, University of Florida, Gainesville, FL, USA; 2 Department of Anatomy and Cell Biology, University of Florida, Gainesville, FL, USA; 3 Department of Biochemistry and Molecular Biology, University of Florida, Gainesville, FL, USA

## Abstract

Adeno-associated virus (AAV) is one of the most promising gene therapy vectors and is widely used as a gene delivery vehicle for basic research. As AAV continues to become the vector of choice, it is increasingly important for new researchers to have access to a simplified production and purification protocol for laboratory grade recombinant AAV. Here we report a detailed protocol for serotype independent production of AAV using a helper-free HEK293 cell system followed by iodixanol gradient purification, a method described earlier.^1^ While the core principals of this mammalian AAV production system are unchanged, there have been significant advancements in the production and purification procedure that serve to boost yield, maximize efficiency, and increase the purity of AAV preps. Using this protocol, we are able to constantly obtain high quantities of laboratory grade AAV particles (>5 × 10^12^ vg) in a week’s time, largely independent of serotype.

## Introduction

Adeno-assoviated virus (AAV) is a small, non-pathogenic parvovirus that infects humans as well as many other animals, including non-human primates. Hundreds of AAV variants and serotypes have been isolated over the past few decades, giving birth to a diverse collection of AAVs with variable tissue tropism. Because of this diverse tropism, different AAV serotypes can be used to target distinctly different populations of cells, making the virus broadly applicable as a gene therapy vector. In addition, AAV has the ability to stably infect quiescent and replicating cells while eliciting a minimal immune response, further broadening its potential as a gene therapy vector.^2^ Recombinant AAV (rAAV) has been used to treat a number of diseases in animal models, such as Leber congenital amaurosis, Duchenne muscular dystrophy, hemophilia B, and Parkinson’s disease.^3^, ^4^, ^5^, ^6^ Recently, an AAV-based gene therapy treatment for Leber congenital amaurosis was approved for clinical use by the United States Food and Drug Administration (FDA).^7^ AAV also has many uses as a laboratory tool, as it can be used to carry reporter genes, therapeutic genes, or Cas9 endonuclease for gene editing in animal models.^8^, ^9^ Because of these numerous applications, it is important to establish a simplified step-by-step protocol for the production of laboratory grade rAAV.

### rAAV Production Systems

On its own, AAV does not possess the ability to efficiently replicate its genetic material, and it requires the presence of a helper virus. Currently, there are three major helper viruses used in the production of AAV: adenovirus (Ad), herpes simplex virus (HSV), and baculovirus (Bac). Ad and HSV helper AAV production methods utilize mammalian cell lines while the Bac system requires insect cells. Both HSV- and Bac-derived AAV production systems are capable of producing very large quantities of AAV; however, they both require construction of recombinant helper virus constructs expressing the transgene of interest.^10^, ^11^ This can be time consuming and is most beneficial for producing large quantities of a single AAV vector construct. In addition to viral titer, different production systems can yield vectors with varying levels of infectivity. This is because different helper systems produce AAVs with slightly different ratios of viral proteins VP1, VP2, and VP3—a phenomenon observed particularly with the Bac system.^12^ Purification techniques and buffer composition can play a role in AAV infectivity as well. In a laboratory setting, Ad-based helper systems are much more cost effective and practical because changes in the transgene construct can be implemented at the plasmid level. This is because, unlike the HSV and Bac system, Ad-based systems are available in a “helper-free” context, i.e., the genes required for helper function have been identified and are expressed via a plasmid, eliminating the need for actual Ad preps to be produced.^13^, ^14^

For these reasons we recommend using the “helper-free” AAV production protocol described here, which requires only three plasmids: one containing the Ad E2A, E4, and VA RNA helper genes (pHelper), another expressing the AAV2/5 rep/cap genes (pACG2R5C), and lastly, the transgene construct flanked by AAV2 inverted terminal repeats (ITRs) (pTR-UF24).^14^, ^15^ It is critical that only the transgene plasmid contains the AAV2 ITRs as these serve as the packaging signal. Note that the plasmids used for this example are specific to the generation of an AAV5-GFP vector and that the AAV5 *cap* gene can be swapped with the *cap* gene from any AAV serotype to create “pseudotyped” vectors.^16^ The transgene plasmid may be exchanged to create vectors expressing any gene of interest. Using these three plasmids, HEK293 cells are triple transfected and used as a production line for rAAV. HEK293 cells are specifically required for the helper-free method as they stably express the Ad E1A/E1B genes which, in combination with pHelper, provide full Ad helper function.^13^ This is the most flexible method for producing rAAV as any changes in serotype or transgene can be made by editing the plasmid constructs and do not require the construction of specific helper viruses.

### Transfection Methods

A key step in the AAV production method is the transfection of HEK293 cells with the Ad helper, AAV rep/cap, and transgene plasmids. It is important to use a practical method that offers both high efficiency and reproducibility. For this reason, we recommend using a polyethylenimine (PEI)-based method as described below. There are alternate transfection methods, such as calcium phosphate or liposomes, that may be used in place of PEI if desired. However, it is important to note that calcium phosphate methods are subject to significant batch variability due to both reagent purity and pH sensitivity. Liposome transfections are highly efficient and minimally cytotoxic, but reagents are much more costly, particularly when used at the scale required for AAV production. Importantly, PEI is also sensitive to changes in pH and, therefore, each new batch should be tested before use to determine the transfection efficiency and cytotoxicity.

### Purification Methods

AAV purification techniques can be separated into two distinct categories, namely density gradient centrifugation or chromatography purification. Both methods, in combination, may be applied to the purification of a single AAV preparation to ensure the purity of the final product. In most cases, however, one method is sufficient to purify laboratory grade rAAV, for which we recommend purification by iodixanol density gradient ultracentrifugation. Iodixanol gradient purification of AAV was first proposed by our group in 1999 and is still one of the most common purification methods used today.^1^ Iodixanol purification is more affordable than affinity and ion exchange chromatography and is serotype independent, minimizing variance between different AAV preps. While iodixanol density purification results in very clean AAV preps, there are, however, known contaminants, namely ferritin, that are still present in the final prep.^17^ If the presence of ferritin interferes with the intended research, it is recommended that additional methods of purification be used.

## Materials

### Reagents

Low passage HEK293 cells (less than p.30, ATCC CRL-1573)
DMEM-high glucose (Sigma-Aldrich cat no. D5796)
Antibiotic-antimycotic 100X (ThermoFisher cat no. 15240062)
Fetal bovine serum (ThermoFisher cat no. 10437036)
Disodium phosphate (Sigma-Aldrich cat no. S7907)
Potassium phosphate monobasic (Sigma-Aldrich cat no. P9791)
NaCl (ThermoFisher cat no. S271)
MgCl_2_ (Sigma-Aldrich cat no. M8266)
KCl (Sigma-Aldrich cat no. P8333)
Optiprep iodixanol solution (Sigma-Aldrich cat no. D1556)
Tris base (ThermoFisher cat no. BP152)
Hydrochloric acid 5M (VWR cat no. BDH7419)
PEG-8000 (ThermoFisher cat no. BP233)
Sodium deoxycholate (Sigma-Aldrich cat no. D6750)
Pierce universal nuclease for cell lysis (ThermoFisher cat no. 88702)
Lactated Ringers (LR) solution (Patterson Veterinary cat no. 07-869-6319)
PEI-MAX, dilute in water to create 40 μm stock solution (Polysciences cat no. 24765-1)
Adenoviral helper plasmid, pHelper (Agilent Technologies cat no. 240071-54)
AAV rep/cap plasmid (pACG2R5C^14^ was used for this experiment and contains AAV2 rep and AAV5 cap sequence)
Transgene plasmid (any gene expression construct flanked by AAV ITRs; a GFP reporter construct was used for this example)

### Equipment

Optiseal polypropylene centrifuge tubes (32.4 mL, 26 × 77 mm) (Beckman Coulter cat no. 361625)
Disposable glass microcapillary pipets, 100 μL (ThermoFisher cat no. K71900-100)
Peristaltic pump PVC tubing (ThermoFisher cat no. 14-283-104)
Conical tubes, 50 mL (ThermoFisher cat no. 14-432-22)
Conical tubes, 250 mL (Corning cat no. 430776)
SafeSeal microcentrifuge tubes, 1.5 mL (VWR cat no. 89082-332)
Needles, 18Gx × 1-1/2” (ThermoFisher cat no. 14-840-97)
Syringe, 5 mL (ThermoFisher cat no. 14-817-29)
Sterile syringe filter, 0.22 μm (CellTreat cat no. 229746)
Watson-Marlow variable speed peristaltic pump, model 205S/CA (ThermoFisher cat no. 14-284-102)
Ti70 ultracentrifuge rotor (Beckman Coulter cat no. 337922)
Metal stand and clamp (ThermoFisher cat no. S24250 and S477653Q)
Optima L-90K ultracentrifuge (Beckman Coulter cat no. 365670)
Sterile 150-mm cell culture plates (Sigma-Aldrich cat no. SIAL0599)
Cell scraper (ThermoFisher cat no. 08-100-241)
Avanti J-E centrifuge (Beckman Coulter cat no. 369001)
JS-5.3 centrifuge rotor (Beckman Coulter cat no. 368690)
50 kDa Vivaspin20 protein concentrating columns (Sartorius cat no. VS2032)

### Reagent Setup

HEK293 medium: DMEM containing 10% FBS and 1% antibiotic-antimycotic. Prepare under sterile conditions.
AAV lysis buffer: 150 mM NaCl, 50 mM Tris-HCl pH 8.5, and 2 mM MgCl_2_ in water
AAV precipitation solution: 40% PEG-8000 and 2.5 M NaCl in water
25% (w/v) sodium deoxycholate in water
Stock solutions for iodixanol gradient purification
10× PBS-MK: 1.37 M NaCl, 80 mM disodium phosphate, 20 mM potassium phosphate monobasic, 10 mM MgCl_2,_ and 27 mM KCl in water
5 M NaCl stock solution
60% iodixanol (Optiprep)
Iodixanol fraction contents: make by diluting 10× PBS-MK, 5 M NaCl, and 60% iodixanol stock solutions into water to reach the final concentrations listed below.
15% Iodixanol fraction (5.5 mL per gradient): 15% iodixanol and 1 M NaCl in 1× PBS-MK
25% Iodixanol fraction (5.5 mL per gradient): 25% iodixanol in 1× PBS-MK
40% Iodixanol fraction (5 mL per gradient): 40% iodixanol in 1× PBS-MK
60% Iodixanol fraction (5 mL per gradient): 60% iodixanol stock in 0.1× PBS-MK
30% Iodixanol fraction (optional, 5.5 mL per gradient, used for second round of purification only): 30% iodixanol in 1× PBS-MK

### Equipment Setup

#### Setting Up the Mechanical Pump

**NOTE:** The peristaltic pump used contains 8 separate channels to allow filling of multiple gradient tubes at the same time (2 channels will be used simultaneously for this example). However, any peristaltic pump can be used to prepare the iodixanol gradient provided that the flow rate is slow enough not to agitate the gradient. If using a single channel pump, be sure to thoroughly flush the tubes with distilled water to flush out the iodixanol fractions between uses.

**CAUTION:** To prevent sample contamination, virus should never be run through the peristaltic pump tubing!

Plug in the peristaltic pump and attach the tubing to the channels being used. Each 26 × 77 mm ultracentrifuge tube will hold just over 10 mL of lysate, so, for this example, we used 2 ultracentrifuge gradients to purify a total of ∼20 mL of crude lysate. Set the pump to run at 25 rpm, and place microcapillary glass pipets on both ends of each tubing (4 total). Fill a beaker with distilled water and place the entrance of each glass pipet in the water. Turn on the pump to be sure that liquid is flowing through all the tubes at relatively the same rate. (If the rate needs to be adjusted for individual channels, turn the adjustment knob until the flow rate for each channel is the same.) Flush all liquid out of the pump channels and pause the pump flow.

## Procedure

### Production of rAAV in HEK293 Cells

1. Propagate HEK293 cells in 15 cm cell culture dishes to produce a total of 10 plates with a cell density of about 75%. Each plate should contain 20 mL of HEK293 medium at all times.
2. Transfect HEK293 cells with all three plasmids (pHelper, pACG2R5C, and pTR-UF24) at an equimolar ratio, detailed in steps 3–10.
3. Prepare two 50 mL conical tubes to be used for the transfection. Label one PEI-MAX and the other DNA.

**NOTE:** The reported values below are for the transfection of a single 15 cm plate of HEK293 cells. When performing the transfection using more plates, simply scale the reagents up to the total number of plates being used. For example, a total of 10 plates were used in this experiment for the production of rAAV5-GFP.

4. To prepare the DNA tube, dilute equimolar amounts of pHelper, pACG2R5C, and pTR-UF24 into DMEM to reach 30 μg plasmid DNA in a total volume of 750 μL.
5. To prepare the PEI-MAX tube, dilute PEI-MAX stock solution into DMEM to a final concentration of 60 μL PEI in a total volume of 750 μL.
6. Vortex both the PEI-MAX tube and the DNA tube and add the contents of the PEI-MAX tube to the DNA tube. Vortex the mixture and let stand for 10 minutes at 37°C.
7. Vortex mixture once more, then add 1.5 mL drop-wise to each 15 cm plate of HEK293 cells, taking care not to disturb the cells.
8. Rock each plate and place in cell culture incubator for 4–6 hr.
9. After 4–6 hr of incubation, remove the transfection media from the HEK293 cells and replace each plate with 20 mL of fresh HEK293 medium.
10. Let transfected cells incubate for 72 hr before harvesting. If you are using a fluorescent reporter transgene plasmid, you should observe close to 75% transfection efficiency by the end of the 72-hr incubation.

### Purification of rAAV from HEK293 Cells

**NOTE:** When purifying rAAV from HEK293 cells, it is important to note that most AAV serotypes will be present both in the cells and in the medium. Therefore, to maximize yield, the virus should be purified from cell culture medium as well. If purifying AAV serotype 2, however, the majority of the virus is associated with the cells, so the medium can be discarded. In the current protocol, we produced rAAV5.

11. Place transfected HEK293 cells in the cell culture hood, remove the lid, and place upside down in the hood.
12. Using a serological pipet, remove ∼10 mL of media from the plate and place into the upside-down 15 cm lid. There should be ∼10 mL of media remaining in the plate along with the cells.
13. Use a cell scraper to detach the cells from the bottom of the plate.
14. Then, using a serological pipet, collect the cells and transfer to a 250 mL conical tube on ice.
15. To ensure that the maximum number of cells are collected, pipet up the 10 mL of media from the lid of the 15 cm plate. Then use this media to thoroughly wash any remaining cells from the plate and transfer to the 250 mL conical tube.
16. Repeat this process for all transfected plates, keeping in mind that a single conical tube can hold the cells and media from about ten, 15 cm plates.
17. Spin cell suspension down at 2,000 rpm for 10 minutes at 4°C to pellet the cells.
18. Carefully pour supernatant into fresh 250 mL conical tubes and store at 4°C for further purification of AAV from cell culture medium (see step 23).
19. Resuspend each cell pellet in 8 mL of AAV lysis buffer, combine into a 50 mL conical tube, and place on ice.
20. Wash each 250 mL tube with 2 mL of AAV lysis buffer to ensure that all cells are collected and combine into a 50 mL conical tube.
21. Freeze the cell suspension at −80°C, then thaw at 37°C in a water bath to lyse cells. Repeat this process twice for a total of 3 freeze and thaw cycles.

**CAUTION:** Once lysate is thawed, place immediately back at −80°C. Excess storage of the lysate at 37°C can degrade the virus.

22. Store the crude lysate overnight at −80°C while the virus is precipitated from supernatant. Crude lysate can be stored indefinitely at −80°C if needed.
23. If purifying AAV from cell culture medium, add 1:4 volume of AAV precipitation solution to the supernatant (generated in step 18), invert tubes to mix, then store overnight at 4°C to precipitate proteins.
24. After precipitation, centrifuge the precipitate at 3,000 rpm for 30 min at 4°C.
25. Discard supernatant.
26. Centrifuge briefly (∼30 s) to collect any excess liquid clinging to the walls of the conical tube, then remove by vacuum aspiration. Take care not to aspirate the pellet.
27. Use 8 mL AAV lysis buffer per conical tube to resuspend the protein pellets, and combine this with the crude lysate.
28. Wash the conical tubes with an additional 2 mL AAV lysis buffer to ensure that all of the pellet is transferred and again combine with the crude lysate.
29. Place the AAV lysate/precipitate mixture in a 37°C water bath and add DNase at a 1:10,000 ratio to digest cellular DNA. Incubate for 30 minutes at 37°C.
30. Add 25% sodium deoxycholate at a 1:50 ratio to the mixture, invert to mix, and incubate for 30 additional min at 37°C. This helps to solubilize precipitate.
31. Spin down cell debris at 4,000 rpm for 15 min at room temperature and transfer supernatant to clean 50 mL conical tube. There should now be roughly 20 mL crude lysate that is now ready for iodixanol density gradient purification.

### Iodixanol Density Purification of rAAV from Crude Lysate

**NOTE:** Before attempting to prepare iodixanol gradients, make sure that all iodixanol fraction solutions are prepared fresh and that there is sufficient volume for the number of gradients required.

32. Using a serological pipet, slowly dispense 10 mL crude lysate into each 26 × 77 mm ultracentrifuge tube.

**CAUTION:** Do not allow any bubbles to form!

33. Iodixanol fractions are then underlaid in the following order using the multichannel pump: 15%, 25%, 40%, and 60%. Each fraction should be in a separate 50 mL conical tube and prepared fresh each time. To fill two gradients, the following volumes of each respective fraction will be used: 11 mL of 15%, 11 mL of 25%, 10 mL of 40%, and 10 mL of 60%.
34. To begin laying the iodixanol gradient, place the entrance capillaries of both pump channels into the 15% iodixanol fraction and lay the exits of both capillaries on a Kimwipe.
35. Turn the pump on to 25 rpm as before and fill the tubing with the 15% fraction. Tubes should be filling at roughly the same rate.
36. Once the liquid reaches the end of each capillary, pause the pump, and place the capillary ends into the bottom of each ultracentrifuge tube containing the crude lysate.

**CAUTION:** Make sure there is no air in the tubing before placing it into the lysate. If each channel is not flowing at the exact same rate, the pump may need to be repeatedly toggled between paused and flowing to ensure that each channel has no air present.

37. Turn the flow rate back on and allow the gradient to slowly fill the bottom of the ultracentrifuge tube.
38. Once the last drops of the 15% fraction begin to enter the pump tubing, immediately pause the pump. There should be less than 0.1 mL of 15% fraction left in the conical tube.

**CAUTION:** Do not allow any air bubbles to enter the pump tubing as this will disrupt the gradient! If air bubbles do enter the tube, pause the pump, set it in the reverse direction, and allow the gradient to flow in the reverse direction to remove the air.

39. Take the entrance of the pump tubing out of the 15% fraction and place it into the 25% fraction to add the next layer. Start the pump.
40. Allow the last of the 25% fraction to enter into the pump tubing and then pause the pump and repeat steps 37 and 38 for the 40% fraction, then the 60% fraction.
41. For the last fraction (60%), continue to fill the ultracentrifuge tubes with as much liquid as possible. Only stop the flow just before air bubbles reach the exit of the pump tubing. Once as much of the 60% fraction has flowed into the ultracentrifuge tube as possible, slowly pull the capillaries out of the gradient.

**CAUTION:** Make sure not to overfill the gradient tubes and keep the tubing as still as possible while pulling straight up into the air to not disturb the gradient!

42. Using a 5 mL serological pipet, carefully fill the top of the ultracentrifuge tubes with AAV lysis buffer to ensure that no air remains.
43. Place caps on each of the ultracentrifuge tubes and carry over to the ultracentrifuge, making sure not to disturb the gradient. The gradient is now ready for centrifugation.
44. Slowly load all gradients into a type Ti70 rotor and load the rotor into the ultracentrifuge.
45. Spin the gradients at 69,000 rpm for 1 hr at 18°C.
46. After centrifugation, carefully remove the gradient tubes from the rotor using a pair of needle nose plyers. It is now time to aspirate the 40%–60% interface, which contains the majority of the packaged AAV particles.
47. Use a metal ring stand and clamp to hold the gradient tube steady for aspiration.
48. Attach an 18G needle to a 5 mL syringe and remove the cap from the gradient tube.
49. Push the needle through the wall of the ultracentrifuge tube about 2–3 mm below the 40%–60% interface (Figure 1).

**Figure 1:**
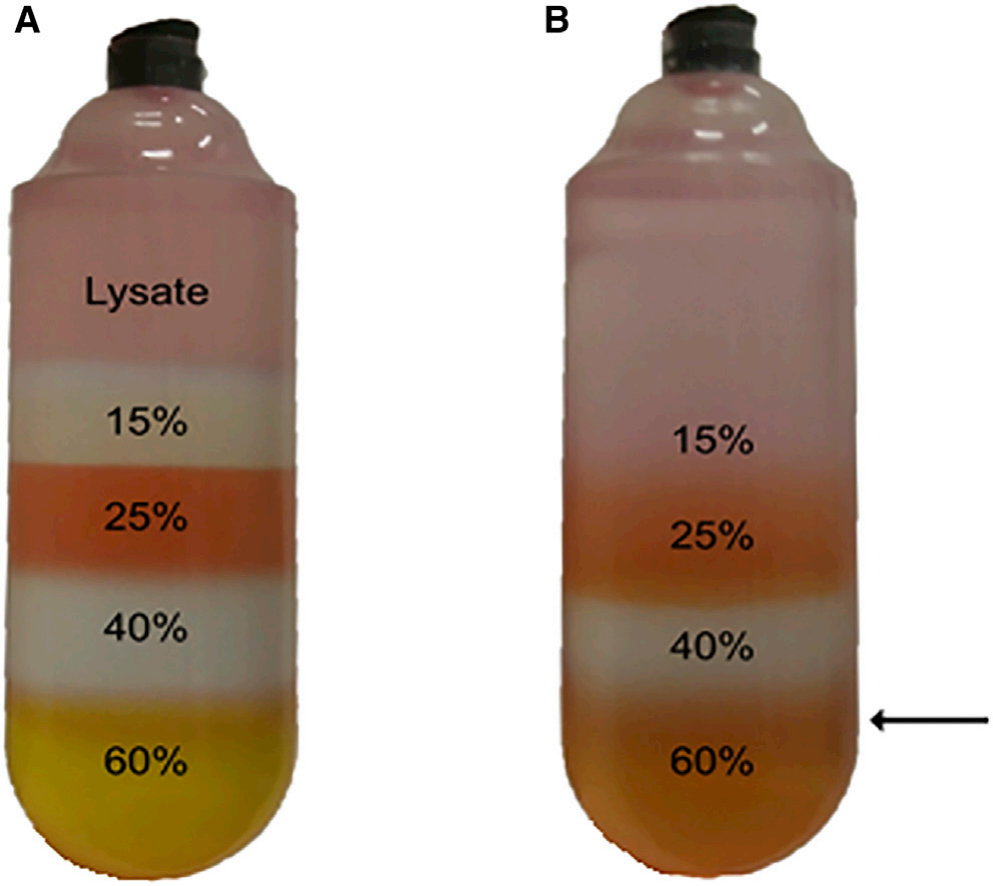
Iodixanol Gradient Centrifugation of AAV Preparation AAV iodixanol density gradient before (A) and after (B) ultracentrifugation. 25% and 60% fractions have 10 ng/mL phenol red added to help with visualization. The black arrow marks the needle injection site for AAV aspiration from the 40%–60% interface.
50. Slowly aspirate the 40%–60% interface until you have 3–4 mL of liquid in the syringe.

**CAUTION:** The top of the 40% fraction contains mostly empty AAV particles, cellular proteins, and deoxycholate (appears as white strands). Make sure not to aspirate any of this along with your fraction!

51. Pull the syringe out of the centrifuge tube and discard the ultracentrifuge tube.
52. Repeat steps 46–50 for all ultracentrifuge tubes and combine all fractions into a 50 mL conical tube (should have about 6–8 mL of total fraction from 2 iodixanol gradients), dilute 1:2 in LR solution, and store on ice. Proceed to the second iodixanol gradient or buffer exchange and concentration.

**NOTE:** We recommend performing additional iodixanol purification after this step to ensure that as much cellular debris as possible has been eliminated from the prep. The cleaner the prep is before concentration, the less likely the chance of unintended precipitation. If working with serotypes that are less prone to precipitation or concentrating to lower titers (10^12^ vg/mL range), one round of iodixanol purification may be sufficient. If choosing not to perform a second iodixanol gradient, procced to Buffer Exchange and Concentration of rAAV.

### Second Lodixanol Gradient Purification (Optional)

**NOTE:** The second round of iodixanol purification is performed identically to the first with the following changes. First, since the density of the sample being purified is now higher than before, the 15% iodixanol fraction is not used. Second, the 25% fraction is replaced with a 30% iodixanol fraction; this is done again because of the increased density of the sample. Third, prior to layering the second gradient, the virus from the first iodixanol gradient should be diluted 2-fold in LR solution to decrease the density of the prep. Because the 15% fraction is omitted, each tube can hold ∼15 mL of sample, allowing for the use of fewer gradient tubes. Other than these changes, the process for laying the gradients, centrifuging the gradients, and aspirating the AAV-containing fraction is the same as single iodixanol purification.

### Buffer Exchange and Concentration of rAAV

53. Equilibrate 50 kDa protein concentration column with 20 mL LR solution and centrifuge at 2,000 rpm and 4°C until all liquid has passed through the column (∼10 min).
54. Discard flow through and top off column with diluted iodixanol fraction obtained in step 52 above.
55. Spin prep at 2,000 rpm for 10 min at 4°C and discard the flow through.

**CAUTION:** It is crucial that the virus is not overconcentrated as this will cause the virus to precipitate out of solution. To avoid this, be sure that after each spin there is at least 1–2 mL of virus remaining in the top of the column. Spin times may have to be adjusted if the fraction flows through the column more rapidly.

56. Repeat steps 54 and 55 until all of the iodixanol fraction has been loaded onto the concentrator column.
57. Wash the column with a total of 50 mL of LR solution to dilute out the residual iodixanol. This will be done by repeatedly centrifuging at 2,000 rpm for 10 min at 4°C, discarding the flow through, and topping off with fresh LR. Be sure that the liquid level remains over 1–2 mL the entire time.
58. After washing, spin the virus down to its final volume. This can be achieved by performing short 2–5 min spins at 2,000 rpm and constantly checking the level using the graduations provided on the column. We recommend aiming for a final volume of 0.5 mL, but, depending on the AAV serotype and intended titer, the virus can be brought down lower to 0.25 mL.

**CAUTION:** The more concentrated the prep is, the more likely precipitation will occur!

59. Once the final volume is reached, use a P1000 pipet to pipet the virus out of the column and place into a low retention microfuge tube.
60. Using a P200 pipet, wash the column with an additional 100 μL of LR to collect any virus clinging to the column wall. Combine this with the virus removed in the previous step and store at −80°C until needed. It is a good idea to save a separate 20 μL aliquot for tittering.
61. It is recommended to sterilize your AAV preparations by passing them through a 0.22 μm syringe filter before long term storage or use in animal models.

**NOTE:** All AAV preps for this example were titered using the serotype-independent PicoGreen method reported by Piedra et al.^18^

### Timelines

Reagent Set-up: ∼1–2 hr
Steps 1–9: ∼4–6 hr
Step 10: 72 hr
Steps 11–22: ∼2–3 hr
Step 23: 24 hr
Steps 24–31: ∼2–3 hr
Steps 32–52: ∼2–3 hr
Second iodixanol purification (optional): ∼2–3 hr
Steps 53–61: ∼3–4 hr

### Troubleshooting

If the final viral preparation has a low titer, there are a few common points in the production and purification process where something may have gone awry. One of the most common causes of low vector yield is poor transfection efficiency. It is important to establish a reliable and reproducible transfection protocol that minimizes cell death and maximizes DNA uptake. If poor transfection efficiency is expected, pilot experiments using a reporter gene and a panel of PEI and DNA ratios should be conducted to establish the ideal experimental parameters for maximum DNA uptake. Another common problem that can result in low vector titer is precipitation of the AAV virus during concentration. This is particularly common when purifying AAV2 or related serotypes and can be prevented by avoiding overconcentration of the viral preparation. If precipitation does occur, however, the viral preparation can be diluted into high salt buffer containing at least 1 M MgCl_2_. The high MgCl_2_ levels will help disrupt protein-protein interactions and resolubilize most of the precipitate. The virus may need to rock overnight to promote dissolving of the precipitate. After resolubilizing the virus, the MgCl_2_ can either be removed via dialysis or buffer exchanged in a protein concentrating column. The virus can then be concentrated into a larger volume to prevent any future precipitation issues.

### Anticipated Results

**Figure 2:**
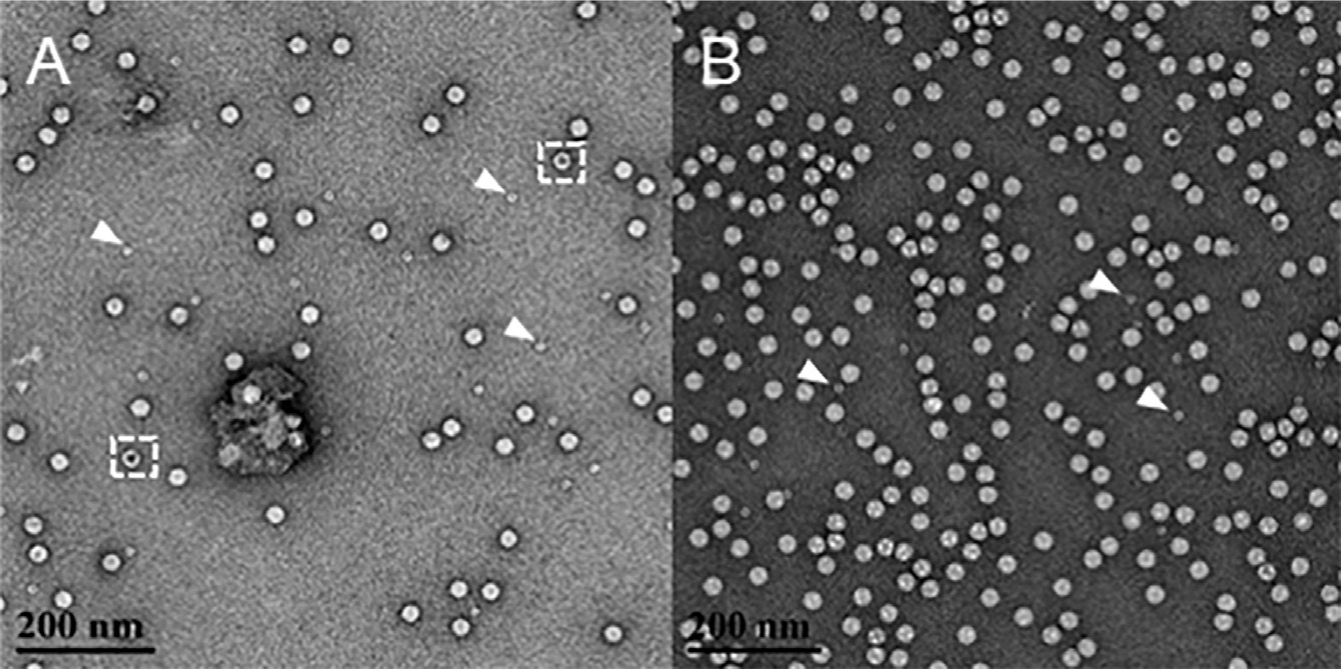
Negative Stain Electron Microscopy Imaging of Final AAV Preparation Negative stain EM image of AAV5 purified by single (A) or triple (B) iodixanol density gradient centrifugation. White arrows denote the presence of ferritin particles, a contaminant from cell lysate not removed by iodixanol density centrifugation. Representative empty AAV particles are inscribed by a dashed white box.

Following this AAV purification protocol will result in rAAV vector yields comparable to those reported in Table 1 below. Certain AAV serotypes tend to produce higher yields than others, so altering the number of cells used for virus production may be required to achieve the intended viral yield. For example, we purified both AAV2 and AAV5 serotypes using this protocol and used double the number of HEK293 cells to produce an AAV2 prep of comparable titer to the AAV5 prep. This is expected as production of the AAV2 serotype is known to produce lower yields (Table 1). Figure 2 shows negative stain electron microscopy (EM) imaging of the AAV5 prep after both single (Figure 2A) and triple (Figure 2B) iodixanol purification. It is evident by EM that iodixanol gradient purification results in clean viral preps, highly enriched for packaged AAV particles. As indicated previously, contaminating ferritin molecules will not be removed by iodixanol purification alone, but this should not interfere with the infectivity of the virus (ferritin is denoted by white arrows in Figure 2).

**Table 1:** PicoGreen Assay Titer Results of AAV2 and AAV5 Double Iodixanol Gradient Purified Preparations

Serotype	No. of 15 cm Plates Used	Titer (vg/mL)	Final Volume (μL)	Yield (vg)
AAV2	20	1.27 × 10^13^	750	9.52 × 10^12^
AAV5	10	1.48 × 10^13^	500	7.40 × 10^12^

As discussed, there are many different purification strategies for achieving laboratory grade rAAV preparations. Helper-free HEK293 AAV production followed by iodixanol density gradient purification is a quick cost-effective strategy that gives high yield purified rAAV preps independent of serotype.

## Author Contributions

P.D. and S.M.C. conducted the experiments that are presented in the anticipated results section of the manuscript. J.K.S. generated the electron microscopy images presented in the anticipated results section of the manuscript. S.M.C. was the primary author for the paper, and all other authors, particularly S.Z., contributed to the final editing and assembly of the manuscript.
